# Transplanting Fecal Virus-Like Particles Reduces High-Fat Diet-Induced Small Intestinal Bacterial Overgrowth in Mice

**DOI:** 10.3389/fcimb.2019.00348

**Published:** 2019-10-15

**Authors:** Derek M. Lin, Britt Koskella, Nathaniel L. Ritz, Dongdong Lin, Amanda Carroll-Portillo, Henry C. Lin

**Affiliations:** ^1^Medicine Service, New Mexico VA Health Care System, Albuquerque, NM, United States; ^2^Department of Integrative Biology, University of California, Berkeley, Berkeley, CA, United States; ^3^Mind Research Network, University of New Mexico, Albuquerque, NM, United States; ^4^Division of Gastroenterology and Hepatology, The University of New Mexico, Albuquerque, NM, United States

**Keywords:** bacteriophage, gut virome, gut microbiome, fecal microbiota transplantation, small intestinal bacterial overgrowth, dysbiosis, high fat diet, microbial therapy

## Abstract

Fecal microbiota transplantation (FMT) is an effective tool for treating *Clostridium difficile* infection in the setting of dysbiosis of the intestinal microbiome. FMT for other forms of human disorders linked to dysbiosis have been less effective. The fecal microbiota contains a high density of virus-like particles (VLP), up to 90% of which are bacteriophages, thought to have a role in regulating gut bacterial populations. We hypothesized that transplantation of the phage-containing fecal VLP fraction may reduce bacterial density in the dysbiotic setting of small intestinal bacterial overgrowth (SIBO). In an experiment using fecal transplantation, we compared the effect of the fecal VLP fraction (bacteria removed) against “Whole” FMT (bacteria intact) on the ileal microbiome. Recipients were either treated with a 30-day high-fat diet (HFD) as a model of dysbiosis to induce SIBO or were on a standard diet (SD). We observed that transplantation of fecal VLPs from donors on a HFD was sufficient to alter the ileal microbiota, but the effect was dependent on diet of the recipient. In recipients on a HFD, ileal bacterial density was reduced. In recipients on a SD, the ileal microbiome transitioned toward the composition associated with a HFD. In both recipient groups, transplantation of fecal VLP fraction alone produced the same outcome as whole FMT. Neither treatment altered expression of antimicrobial peptides. These findings demonstrated a potential role of VLPs, likely phages, for modifying the gut microbiome during dysbiosis.

## Introduction

During fecal microbiota transplantation (FMT), a large population of virus-like particles (VLPs) are transferred into the gastrointestinal tract of the recipient. There is an estimated population of 10^9^ VLPs per gram of feces, a population density equal to that of fecal bacteria (Kim et al., 2011). Bacteriophages (phages) make up ~90% of VLPs in a healthy gut virome (Reyes et al., 2012) and have been observed to modulate the composition, structure, and function of gut microbial communities (Abeles and Pride, 2014; Kim and Bae, 2016; Bao et al., 2018; Hsu et al., 2018; Moreno-Gallego et al., 2019). The gut phage community has also been associated with microbial “dysbiosis” (herein referred to as a microbiome state significantly associated with decreased health) in such disorders as Crohn's Disease, ulcerative colitis, and colorectal cancer (Pérez-Brocal et al., 2013; Wagner et al., 2013; Norman et al., 2015). Whether phages have a direct role in causing or preventing dysbiosis is unknown but the possibility is both an important concern with regards to existing FMT treatments and promising implication for future microbiome-targeting therapies.

Small intestinal bacterial overgrowth (SIBO) is a form of dysbiosis characterized by increased colonization of the small intestinal mucosa by resident bacteria (Lin, 2004). SIBO may be induced by a variety of disruptors of the gut including a high-fat diet (HFD) (Tomas et al., 2016) and is commonly treated with antibiotic therapy. This approach is not always effective and recurrence of SIBO is common (Lauritano et al., 2008; Pimentel et al., 2009). Phage therapy is an alternative antibacterial treatment option that has not yet been investigated for SIBO. Historically, strain-specific phage therapy has been used to kill a targeted bacterial pathogen at a specific site of infection (Lin et al., 2017). It is not known whether a diverse population of phages, such as that represented in the fecal VLP fraction could be used to reduce colonization by a diverse population of resident bacteria across a large surface area such as the small intestinal mucosa.

The present study is an investigation into the impact of donor-derived fecal VLPs on the gut microbiome of recipients during FMT. Donor mice were treated with a HFD, and their feces were transplanted either whole (FMT) or processed to remove bacteria (VLP) into the gut of recipient mice that were fed either a HFD or a standard diet (SD). Specifically, we tested the hypothesis that transplantation of an extracted fraction of fecal VLPs may reduce bacterial density of the small intestinal mucosa by comparing the effects of these two transplants (FMT vs. VLP) on mucosa-associated bacterial density and gut microbiome composition.

## Methods

### Animals

**Figure 1:**
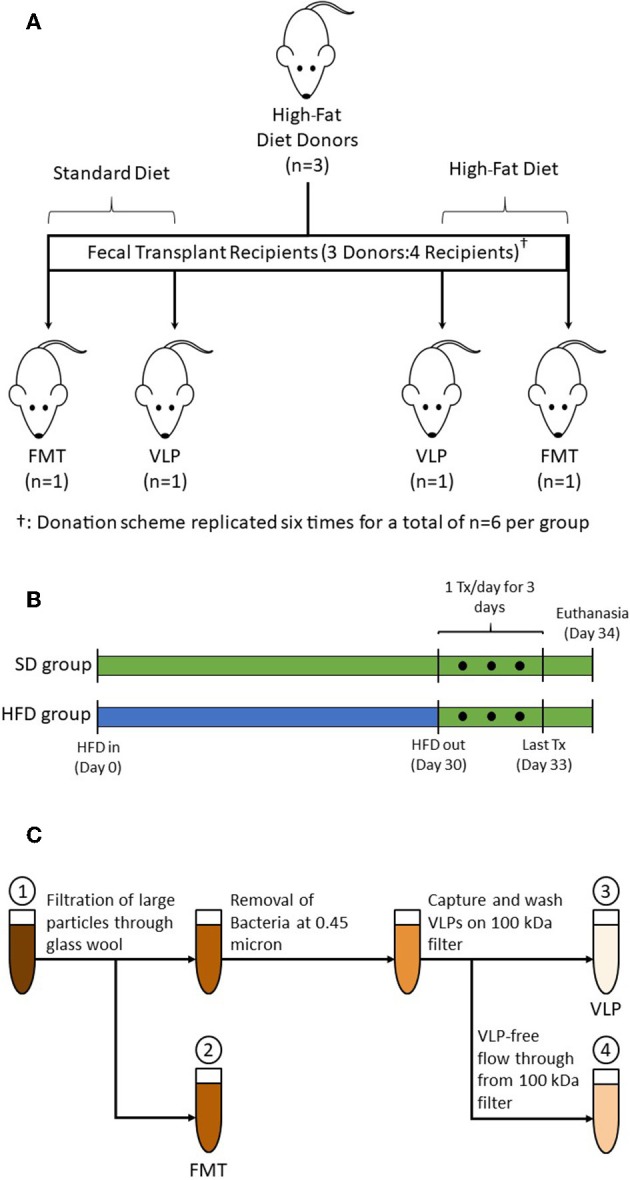
**(A)** A total of 18 donor mice were randomized into subgroups of *n* = 3, each subgroup (one shown as representation) provided transplants for four recipient mice. Fresh pellets from each subgroup were collected, pooled, and processed into treatments: one each of HFD-FMT, HFD-VLP, SD-FMT, and SD-VLP. This donation scheme was replicated six times for total recipient group sizes of *n* = 6. Additional groups of mice receiving PBS control, SD-PBS and HFD-PBS, are not pictured. **(B)** HFD groups were treated with 30 days of HFD, at which point fecal samples were collected from donors. After 30 days on the HFD, mice were transitioned back to a SD for 24 h prior to receiving treatments which were administered once daily for 3 consecutive days. Mice were euthanized 24 h after final treatment. SD groups followed the same schedule but remained on a SD for the duration of the experiment. **(C)** Processing steps for whole FMT and extracted fecal VLP treatments: (1) Homogenized fecal pellets in PBS; (2) Fecal suspension whole fecal microbiota for FMT; (3) Bacteria-depleted, extracted fecal VLP fraction for VLP treatments; and (4) Fecal suspension of sub-viral components (filtered at ~3 nm).

The experimental procedures and animal use were approved by the Institutional Animal Care and Use Committee at the New Mexico VA Health Care System following guidelines provided by the Guide for the Care and Use of Animals of the National Research Council of the National Academies. A total of thirty-six 8-week-old C57BL/6 mice (Charles River, Wilmington, MA) were used. Mice were randomized into two dietary groups (*n* = 18 each): SD or HFD (Figure 1A). Mice from each dietary group were further randomized into three treatment groups: phosphate-buffered saline (PBS) control, FMT, or VLP. The recipient groups are named according to their “Diet-Treatment” (*n* = 6 each): SD-PBS, SD-FMT, SD-VLP; HFD-PBS, HFD-FMT, HFD-VLP. All HFD mice were given a 30-day treatment of a custom HFD (TD.170686, Envigo, Madison, WI; 40% lard by weight) before being transitioned to a standard mouse diet (2920X.10, Envigo, Madison, WI) (Figure 1B); SD mice remained on the standard mouse diet for the entirety of the experiment. All recipient mice received 400 μL of their designated treatment via oral gavage into the stomach 24 h after the transition of HFD groups to the SD. Treatments were given once daily for 3 days and mice were sacrificed 24 h after final treatment at which point tissues were collected, gently washed to remove luminal contents, and stored in 200 proof ethanol at −80°C until processing. Fecal, not luminal, samples for microbiome analysis were collected and stored in 200 proof ethanol at −80°C prior to sacrifice.

### Fecal Transplant Preparation

Fecal transplants were prepared using fecal samples collected from a total of 18 donor mice on day 30 of their HFD treatment. Donor mice were randomized into subgroups of three (for a total of six subgroups); pooled samples from each subgroup provided transplants for four recipient mice: one each of SD-FMT, SD-VLP, HFD-FMT, and HFD-VLP (Figure 1A). Therefore, in each comparison, the VLP treatment was derived from the same fecal samples used for FMT, and each replicate recipient within a given treatment was biologically independent (i.e., receiving transplants from an independent subgroup). The processing steps involved homogenizing 1.0 g of fresh fecal samples from each subgroup, suspending in PBS, and separating into two aliquots of equal volume: one for processing into a FMT and one for extracting the VLPs (Figure 1C). For FMT treatments, the fecal suspension was minimally processed using only filtration of the fecal suspension through glass wool to remove of large particles and debris. The FMT fraction was used for both SD-FMT and HFD-FMT recipient groups.

VLPs are known to flocculate with particles in activated sludge (Brown et al., 2015), therefore we began VLP extraction by agitating the fecal suspension using 1 mm zirconia/silica beads and a bead beater to dislodge extracellular VLPs from fecal particulate matter. The suspension was then centrifuged at 5,000× g for 10 min to pellet cells and large particles. The VLP-containing supernatant was subsequently filtered through a 0.45-μm filter to remove bacteria (Carter, 1996). The bacteria-depleted fecal filtrate was then filtered further using an Amicon Ultra-4 100 kDa centrifugal filter (Millipore Sigma), which has a pore size small enough to capture all known VLPs (~3 nm; Bonilla et al., 2016). VLPs on the filter were washed 3× with PBS and then resuspended in a volume of PBS equivalent to the starting volume of fecal suspension. The extracted fecal VLP fraction was used for both SD-VLP and HFD-VLP recipient groups. Samples of the extracted VLP fraction and flow through from the Amicon 100 kDa filter were examined using electron microscopy and flow cytometry to confirm absence/presence of phage. All PBS used for processing was filtered with a 0.02-μm pore size to exclude any existing bacteria and VLPs.

All mice in SD-PBS and HFD-PBS groups received a 0.02-μm-filtered PBS blank solution as a control treatment.

### DNA/RNA Extraction

Immediately after collection, sections of the intestine were gently washed with sterile PBS to remove luminal contents and were then stored at −80°C until analysis. Intestinal samples were thawed and two 25 mg sections of tissue were excised from the mid-ileum for either DNA or RNA extraction. Genomic DNA was extracted with the DNeasy Blood and Tissue Kit (Qiagen) and RNA with the RNeasy Mini Kit (Qiagen); RNA was reverse transcribed into cDNA using the SuperScript III First-Strand Synthesis System (Invitrogen). Fecal microbiome DNA was extracted using the QIAamp DNA Microbiome Kit (Qiagen).

### Quantitative Polymerase Chain Reaction (qPCR)

We used qPCR to assess the overall bacterial density of the small intestinal mucosa by quantifying copies of the universal 16s rRNA gene in extracted genomic DNA with the Eub338/518 primer pair. Primer pairs can be found in Table 1. All reactions were completed with the Quantitect SYBR Green qPCR Mastermix (Qiagen). Calculations were performed with the ΔΔCt analysis using the universal eukaryotic 18s rRNA gene for standardization. All qPCR results are reported as fold-change (mean ± SEM) relative to the SD-PBS Control group.

**Table 1:** List of primer pairs used for qPCR analysis.

	**Forward (5^**′**^ to 3^**′**^)**	**Reverse (5^**′**^ to 3^**′**^)**
18s	GTAACCCGTTGAACCCCAT	CCATCCAATCGGTAGTAGCG
16s	ACTCCTACGGGAGGCAGCAG	ATTACCGCGGCTGCTGG

### 16s Amplicon Sequencing

All library preparations and sequencing runs were performed on a fee basis by the University of Minnesota Genomics Center (genomics.umn.edu). Extracted genomic DNA samples from the ileum were used as templates for PCR amplification of the V5-V6 region of the 16s rRNA gene. Degenerate primer sets containing Illumina adapters were used for library creation. The primer sequences, with the 16s-specific portion in bold, were as follows:

V5F_Nextera (TCGTCGGCAGCGTCAGATGTGTATAAGAGACAG**RGGATTAGATACCC**); V6R_Nextera (GTCTCGTGGGCTCGGAGATGTGTATAAGAGACAG**CGACRRCCATGCANCACCT**). To add the indices and flow cell adapters, the following indexing primers were used: Forward indexing primer (AATGATACGGCGACCACCGAGATCTACAC[i5]TCGTCGGCAGCGTC); reverse indexing primer (CAAGCAGAAGACGGCATACGAGAT[i7]GTCTCGTGGGCTCGG). The p5 and p7 flow adapters are in bold and [i5] and [i7] refer to the index sequence codes used by Illumina. All PCR reactions were performed using KAPA HiFidelity Hot Start Polymerase (KAPA Biosystems, KK2103). The initial PCR reaction had the following conditions: 95°C for 5 min and 25 amplification cycles (98°C for 20 s, 55°C for 15 s, 72°C for 1 min). PCR products were diluted 1:100 and 5 μL were used in the second PCR reaction that had the same conditions, albeit with 10 cycles. Indexed PCR product was quantified using PicoGreen (ThermoFisher, P11496) equal-volume normalized, and 10 μL of each sample was pooled. The pooled PCR product was denatured with NaOH, diluted to 8 pM in Illumina's HT1 buffer, spiked with 15% PhiX, and heat denatured at 96°C for 2 min prior to loading. A MiSeq 600 cycle v3 kit was used to sequence the sample. For post-run trimming, the following Nextera adapter sequences were used: Read 1 (CTGTCTCTTATACACATCTCCGAGCCCACGAGACNNNNNNNNATCTCGTATGCCGTCTTCTGCTTG); Read 2 (CTGTCTCTTATACACATCTGACGCTGCCGACGANNNNNNNNGTGTAGATCTCGGTGGTCGCCGTATCATT).

### 16s rRNA Sequencing and Analysis

Raw sequencing data were demultiplexed and quality controlled by applying the pipeline in DADA2 to generate a number of unique sequences. Each sequence was trimmed by the length of 260 base pairs for forward direction and 220 bps for reverse direction based on quality score of >30. The remaining reads were aligned by the mafft tool to build a phylogenetic tree. Rarefaction of raw reads occurred at 5,000 sequences/sample. A total of 13 mice were excluded from analysis due to low abundance after rarefaction: one from HFD-PBS, two each from SD-PBS, SD-FMT, SD-VLP, and HFD-FMT, and four from HFD-VLP. Alpha diversity metrics (Chao1, Simpson, Shannon, and observed OTUs) and beta diversity (weighted UniFrac) were assessed for all remaining individuals in each group. All the processing scripts were implemented on a QIIME2 (https://qiime2.org/, 2017.12 release) platform.

### Flow Cytometry

Extracted fecal VLP samples were analyzed with flow cytometry to visualize the extracted VLPs. Both the extracted VLP solution and the flow through from the Amicon Ultra-4 100 kDa centrifugal filter were prepared for flow cytometry according to an established protocol (Brussaard, 2009). Briefly, the samples were diluted to 1:1,000 and stained using a commercial stock of SYBR Green I (final concentration of 1:10,000; ThermoFisher, S7567). Fluorescent VLPs were analyzed using an Attune NxT flow cytometer (Thermo Scientific) which was programmed to assess side scatter and fluorescence at a flow rate of 12.5 μL/s for a total of 2 min. Results are reported as average total events (mean ± SEM).

### Transmission Electron Microscopy (TEM)

TEM images were captured in the HSC-Electron Microscopy Facility, supported by the University of New Mexico Health Sciences Center. Briefly, 5–10 μL of the extracted fecal VLP fraction was incubated on glow-discharged, carbon-coated grids for 5 min, grids were then washed 3× in ultrapure H_2_O, excess liquid was wicked away, and the samples were stained for 1-2 min with 1% uranyl acetate. The excess stain was wicked away and grids were left to air dry. Samples were examined in a Hitachi HT7700 TEM operating at 80 kV; images were captured with an AMT XR-81 CCD camera.

### Statistical Analysis

All statistical analyses were performed with consultation by a statistician. All qPCR data and phyla level 16s data were compared using two-way ANOVA analysis with Grubb's test to identify outliers. Multiple comparisons testing was performed using Holm–Sidek correction. These statistical analyses were performed with GraphPad Prism (GraphPad Software). Mouse weights over the course of the experiment were compared using two-way ANOVA analysis with repeated measures and multiple comparisons using Holm–Sidek correction. These analyses were performed on SPSS (IBM Corp.). All alpha and beta diversity measures were compared with Wilcoxon sum-rank and Kruskal–Wallis testing using permutational MANCOVA with 999 permutations. These statistical analyses were performed with the QIIME2 (https://qiime2.org/, 2017.12 release) platform.

## Results

### Validation of Fecal Filtration Steps for Phage Extraction

**Figure 2:**
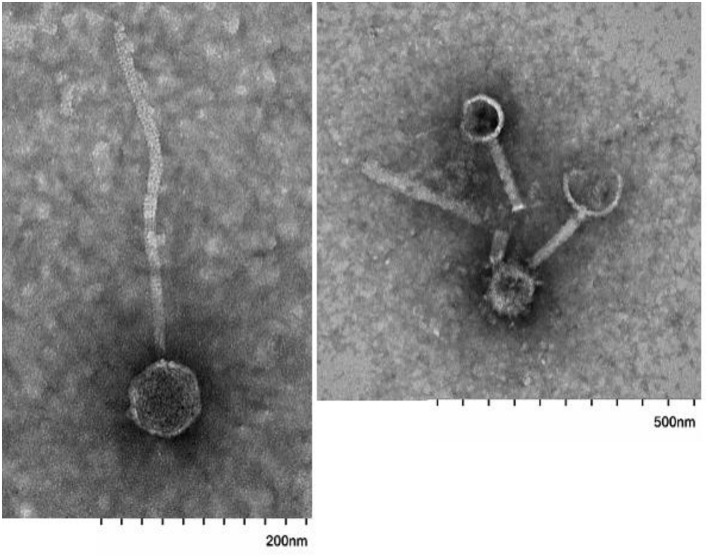
Images of tailed phages in fecal VLP fractions identified using electron microscopy. Scale bars are indicated at bottom.

**Figure 3:**
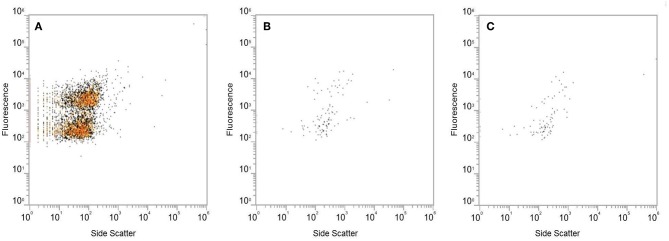
Analysis of the SYBR Green I-stained extracted VLP fraction by flow cytometry, using fluorescence and side scatter as metrics. Level of fluorescence differentiated two populations of VLPs in the extracted VLP fraction **(A)**. The fluorescent signal produced by the flow through from Amicon Ultra 100 kDa filters **(B)** was at the same level as the PBS blank **(C)**.

Analysis of EM images identified several phages with diverse morphology in the extracted fecal VLP fraction (Figure 2). All clearly identifiable phages were tailed, characteristic of the order *Caudovirales*. In this analysis, there were no bacteria present, confirming the depletion of bacteria using filtration There was also no clearly identifiable non-phage VLPs, which is appropriate given the known predominance of phages in the fecal VLP fraction. Analysis of the SYBR Green I-stained VLP fraction using flow cytometry identified two distinct populations of VLPs, which could be differentiated based on level of fluorescence (Figure 3). Total events in 1.5 mL of extracted fecal VLP at a 1:1,000 dilution were significantly higher than in the flow through from 100 kDa filtration units (8415.7 ± 347.7 and 167 ± 50.5, respectively, *p* < 0.05) and the flow through was not significantly different from a PBS blank (142.7 ± 35.8; *p* = 0.9). It should be noted that flow cytometry for viral enumeration is limited by significant background noise and these counts are not intended for quantitation but instead, to qualitatively demonstrate the presence of VLPs in the VLP fraction and absence in the flow through. No phages were found in the EM imaging of the flow through corroborating the finding from flow cytometry. These findings based on the “Phage-on-Tap” protocol by Bonilla et al. (2016) demonstrated that a combination of centrifugation and size filtration could effectively fractionate the fecal microbiota into a sub-bacterial fraction, which removes bacteria but retains VLPs, and a sub-viral fraction, which further removes VLPs.

### HFD Dysbiosis Model

**Figure 4:**
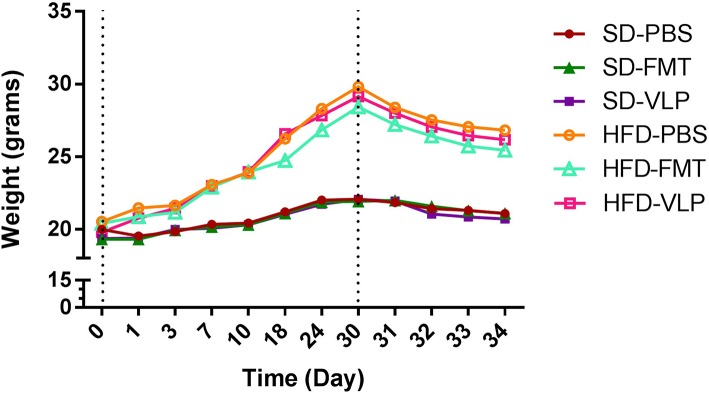
Mean weight change for each group over the course of the experiment represented in grams.

Mice rapidly gained weight in response to the HFD. Two-way ANOVA analysis revealed a main effect of diet [*F*_(1, 30)_ = 44.712, *p* < 0.0001], but no effect of treatment [*F*_(2, 30)_ = 0.213, *p* = 0.8] and no interaction between diet and treatment [*F*_(1, 30)_ = 0.151, *p* = 0.8]. By day 30, HFD mice were significantly greater in weight compared to SD mice (HFD = 29.1 ± 0.6 g; SD = 22.0 ± 0.4 g) (*p* < 0.0001) (Figure 4). All HFD mice, regardless of treatment began to lose weight once they were transitioned back to a SD but by day of euthanasia, the HFD group was still significantly greater in weight compared to the SD group (HFD = 26.1 ± 0.6 g; SD = 20.95 ± 4 g) (*p* < 0.0001).

### Bacterial Density of the Ileal Mucosa

**Figure 5:**
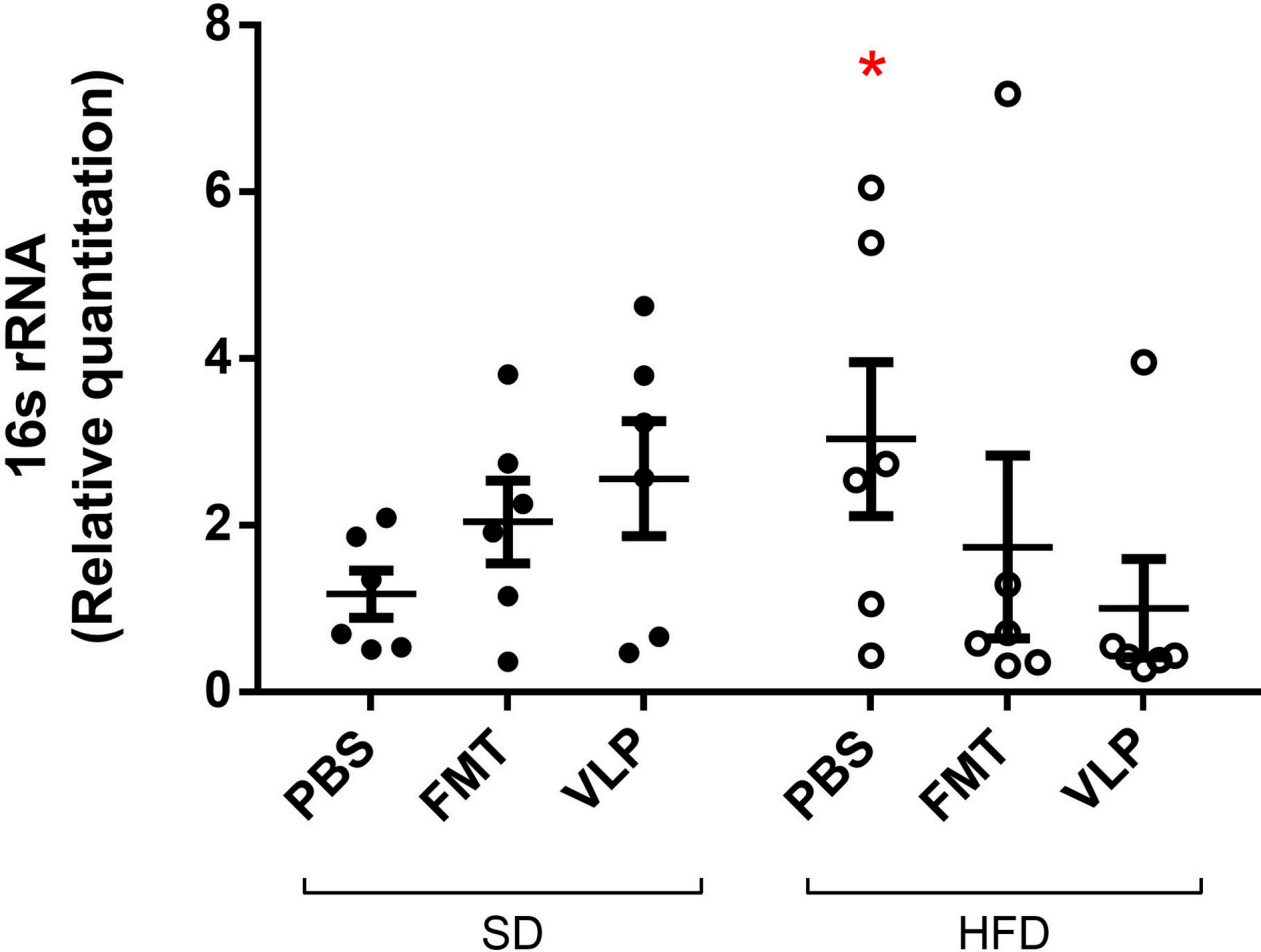
Relative quantitation of the bacterial 16s rRNA gene as a measure of mucosa-associated bacterial density. Data collected by qPCR analysis of extracted DNA from ileum of the small intestine are represented as fold-change relative to the SD-PBS control group and presented as mean ± SEM. *Post-hoc* analysis of these data using Grubb's test identified two outliers >2 standard deviations from the mean (*p* < 0.001), one from the HFD-FMT group and one from the HFD-VLP group. Data from these individuals were included in the graph but removed from all analyses (^*^p < 0.05).

As a measure of mucosa-associated bacterial density, qPCR was used to compare relative quantitation of the bacterial 16s rRNA gene in genomic DNA extracted from ileal tissue. Two-way ANOVA analysis found a significant interaction effect between type of treatment and recipient diet on bacterial density of the ileal mucosa [*F*_(2, 28)_ = 7.3, *p* < 0.005), but no main effect of diet or treatment. The ileum of the HFD-PBS group had on average a three-times increase in bacterial density (HFD-PBS = 3.0 ± 0.9) relative to the SD-PBS control group (SD-PBS = 1.2 ± 0.3; *p* < 0.05) confirming that HFD induced small intestinal bacterial overgrowth (SIBO). Treatment of HFD mice with either FMT or VLP treatment was associated with a reduced bacterial density relative to the HFD mice receiving a PBS control (HFD-FMT = 0.7 ± 0.2, *p* < 0.05; HFD-VLP = 0.4 ± 0.0; *p* < 0.05). By the end of the experiment, bacterial density of HFD recipients receiving VLP or FMT was statistically no different than that of the SD-PBS control group (both *p* = 0.6; Figure 5). In SD recipients, neither FMT nor VLP treatment had a significant effect on bacterial density compared to SD-PBS controls (SD-FMT = 2.0 ± 0.5, *p* = 0.6; SD-VLP = 2.6 ± 0.7; *p* = 0.3). *Post-hoc* analysis of these data using Grubb's test identified two outliers >2 standard deviations from the mean (*p* < 0.001), one from the HFD-FMT group and the other from the HFD-VLP group. Data from these individuals were included in graphs but removed from analyses.

### 16s Sequencing Results

**Figure 6:**
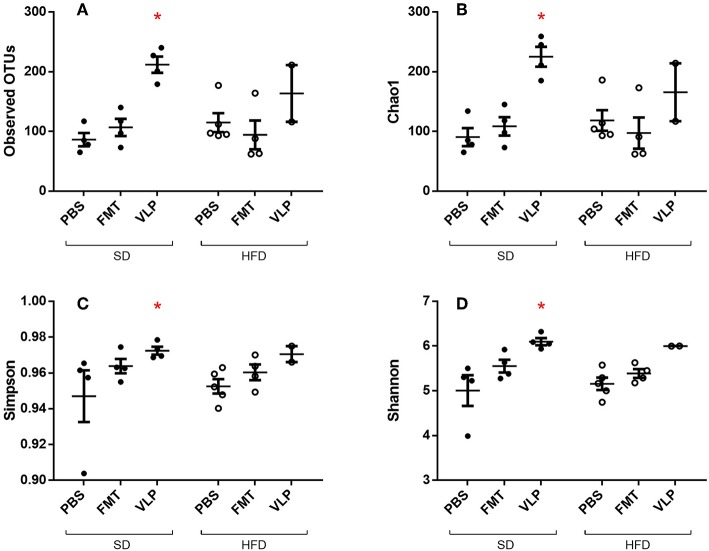
Alpha diversity measures of ileal microbiome as determined by Observed Operational Taxonomic Units (OTU) **(A)**, Chao1 index **(B)**, Simpson index **(C)**, and Shannon index **(D)**. Means with asterisks are significantly different from the SD-PBS control group (^*^p < 0.05).

**Figure 7:**
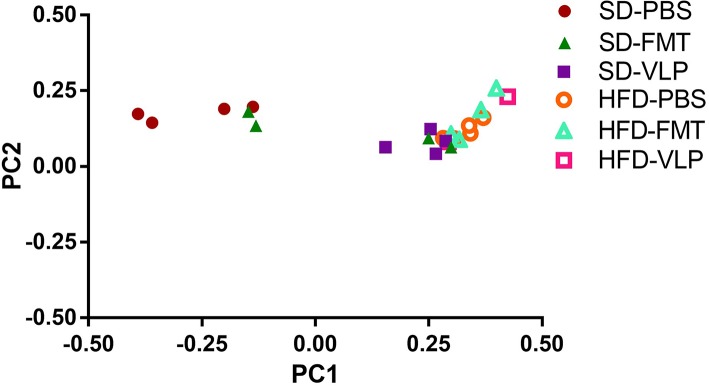
Principal coordinates analysis plot of weighted UniFrac distances for ileal microbiome beta diversity. A total of 13 mice were excluded from analysis due to low abundance after rarefaction: one from HFD-PBS, two each from SD-PBS, SD-FMT, SD-VLP, and HFD-FMT, and four from HFD-VLP.

We sequenced extracted DNA samples from both ileal mucosa and the feces to characterize both ileal and fecal microbiome, respectively. Given our lab's interest in the mucosa-associated bacteria associated with SIBO, we focused on results from the ileal microbiome. Treatment of the SD mice with VLP was associated with an increase in all alpha diversity index measures relative to the SD-PBS control group based on Wilcoxon rank-sum test (all *p* < 0.05; Figure 6). After rarefaction, only two members of the HFD-VLP group remained for analysis, therefore no conclusions could be drawn about changes to diversity resulting from VLP treatment in HFD recipients. Beta diversity analysis using weighted UniFrac measures identified distinct microbial community compositions differentiating the SD-PBS and HFD-PBS groups, as indicated by clustering on the principal coordinates analysis (PCoA) plots (*p* < 0.01; Figure 7). SD mice treated with either FMT or VLP from a HFD donor had a community composition associated with the HFD cluster and were significantly different from SD-PBS control group (SD-FMT: *p* < 0.05; SD-VLP: *p* < 0.05). Interestingly, all individuals in the SD-VLP group clustered with the HFD-PBS group but half the individuals in the SD-FMT treatment group remained with the SD-PBS cluster. The HFD-FMT group also clustered with the HFD-PBS group and was significantly different from the SD-PBS group (*p* < 0.05). These findings suggest that any combination of HFD and/or FMT or VLP from a donor on a HFD was sufficient for promoting a HFD-associated microbiome composition. There were no significant differences between groups based on sequencing data from fecal microbiome DNA.

**Figure 8:**
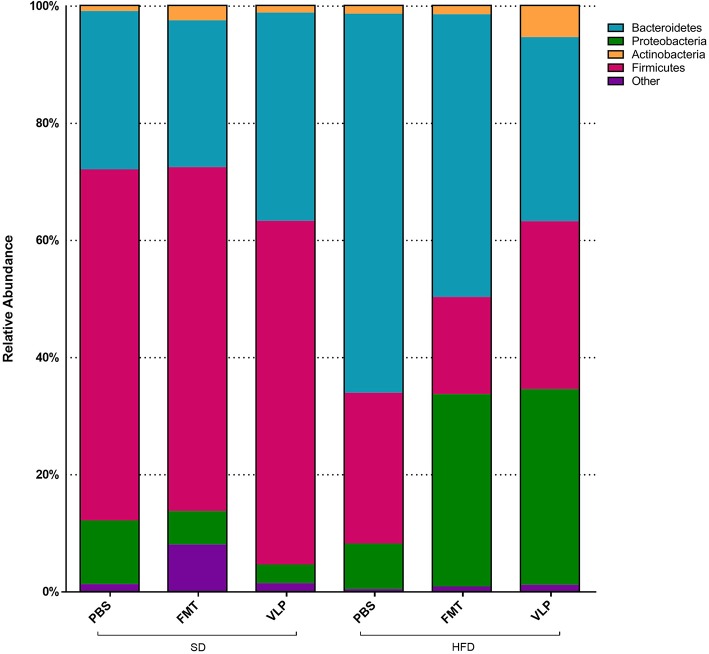
Proportional representation of major bacterial phyla for each dietary-treatment group based on 16s amplicon sequencing results.

At the phyla level, we found a main effect of the HFD associated with increased Proteobacteria relative to the SD group [*F*_(1, 30)_ = 6.041, *p* = 0.02; Figure 8] but no effect of fecal transplant treatment [*F*_(2, 30)_ = 0.7377, *p* = 0.48] and no interaction [*F*_(2, 30)_ = 2.078, *p* = 0.14]. Overall, there was a main effect of HFD associated with a reduction in Firmicutes relative to the SD group [*F*_(1, 30)_ = 6.041, *p* < 0.0001], regardless of fecal transplant treatment, and there was no effect of treatment [*F*_(2, 30)_ = 0.2868, *p* = 0.75] or interaction [*F*_(2, 30)_ = 0.2576, *p* = 0.77]. At this point it is not known why only Proteobacteria, and not Firmicutes, expanded in response to the HFD but it may be associated with the transition back to a SD.

## Discussion

Following induction of SIBO using a 30-day HFD model, we found that bacterial density, as estimated by the relative quantitation of 16s rRNA, was significantly reduced in HFD recipients receiving either VLP or FMT transplants, as compared to HFD-PBS control mice. Specifically, recipients receiving either VLP or FMT, but not PBS, had bacterial densities statistically no different from that of SD-PBS control mice by the end of the experiment. In addition, transplanting either FMT or VLP from a HFD donor into SD recipients shifted their ileal microbiome toward a HFD-associated bacterial community composition. Together these findings suggest fecal VLPs were responsible for the observed effect.

FMT is an established clinical treatment for recurrent *C. difficile* infection (Kassam et al., 2013) and has also been explored as a treatment for other dysbiosis-associated disorders (Vrieze et al., 2012; Colman and Rubin, 2014; Moayyedi et al., 2015; Heath et al., 2018; Gogokhia et al., 2019). The majority of FMT studies have been limited to the role of donor-derived fecal bacteria on the recipient's colonic microbiota (Pamer, 2014). However, in addition to fecal bacteria, FMT transfers a collection of host cells, protists, archaea, VLPs (~90% phages), fungi, and signaling molecules with no understanding of how those components affect the recipient (Reyes et al., 2012; Bojanova and Bordenstein, 2016). A recent clinical study demonstrated the therapeutic potential of these sub-bacterial components by using a fecal suspension filtered to remove bacteria as an effective clinical treatment of *C. difficile* infection (Ott et al., 2017). Understanding how these non-bacterial components interact with resident microbiota throughout the intestine is an essential next step in exploring the potential of FMT-based treatments for various forms of gut dysbiosis. Phages specifically are of interest for treatment of *C. difficile* infection, where successful resolution is associated with uptake of the donor's phage community (Zuo et al., 2018), and whether donor phages become established in the recipient is dependent on donor-recipient pairing (Draper et al., 2018).

In the present study, we found that the outcome of administering a diverse phage population, as found in the fecal VLP fraction, depends on the diet-associated resident microbiota and gut environment of the recipient. One possible explanation for these diet-dependent effects of the VLP treatment lies in the evolutionary theory of local adaptation, where parasites become specialized to hosts from their local “sympatric” environment and thus are less infective to hosts from other environments. Unlike broad-spectrum antibiotics, phages infect a relatively narrow range of bacterial hosts, and previous work has found phages to locally adapt, exhibiting higher infectivity against local strains of bacteria relative to those collected from different locations (Vos et al., 2009; Koskella et al., 2011). A HFD produces characteristic changes to gut microbial community composition, such as an expansion in the relative proportion of Proteobacteria (Hildebrandt et al., 2009). Within the framework of local adaptation, the specialization of HFD-associated gut phages to HFD-associated gut bacteria may account for the divergent responses of HFD- and SD-associated gut bacteria in recipient mice to fecal phages derived from a HFD donor. This hypothesis is supported by the finding that neither FMT nor VLP reduced overall bacterial density in SD recipients. In fact, there were non-significant trends toward increased bacterial density in SD-VLP and FMT groups. The present study is focused on transplantation of HFD-associated VLPs to SD recipients to test whether an induced phenotype change in the donor could be transferred to a recipient using VLPs. Phage-mediated reduction in bacterial density mirrors the outcome of phage therapy, which utilizes strain-targeting phages to eliminate a particular pathogenic bacteria at the site of infection. It follows that administering a diverse population of phages (e.g., the fecal phage population) may eliminate a diverse population of bacteria (e.g., resident small intestinal bacteria). An analogous phage transplant study in a plant model also found a uniform reduction in overall bacterial density immediately after treatment with a diverse phage community, followed by a restructuring of the bacterial community 1-week post treatment (Morella et al., 2018). Our model was limited to a short-term analysis immediately after treatment and it is not known whether these changes will persist. We found that neither VLP nor FMT significantly altered the population density of resident small intestinal bacterial community composition in SD recipients but did transition small intestinal bacterial composition to resemble that of a HFD microbiome. Principal coordinates analysis of weighted UniFrac measures of 16s rRNA sequencing data found that SD mice treated with either FMT or VLP from a HFD donor clustered more with HFD mice than with SD-PBS controls as demonstrated on PCoA plots (Figure 7). This indicates that fecal VLPs alone were sufficient to drive microbiome composition toward that of the donor. The VLP treatment also activated the ileal microbiome in a way that increased alpha diversity of the mucosa-associated bacterial community in SD recipients (Figure 6). The compositional shift toward a HFD-associated microbiota in SD recipients after 3 days of treatment was accompanied by a trend toward increased ileal bacterial density. The observed impact on microbial community composition and diversity suggests a complex interaction between fecal VLPs and gut bacteria that goes beyond simple changes in abundance of bacterial members. While the mechanism of this outcome is outside the scope of this study, one hypothesis involves the phage-mediated horizontal transfer of genes from the HFD- to SD-associated microbiota during FMT or VLP treatment. Kim and Bae (2016) found an expansion of phage-encoded genes for bacterial fitness (e.g., bacterial metabolism) in the gut phageome in response to a HFD. These phages disproportionately target Firmicutes and Proteobacteria (Kim and Bae, 2018), two phyla that expand during HFD dysbiosis. Transfer of these phages into an SD recipient may provide a fitness advantage to resident bacteria in these phyla, allowing for their expansion in a way that changes microbial community composition to resemble the donor community. Future metagenomic analyses of donor and recipient viromes will be necessary to test this hypothesis.

Exogenous phage administration for changing gut microbial community composition has been demonstrated in recent studies (Bao et al., 2018; Hsu et al., 2018). Using a defined community of resident gut bacteria (commensals) in a gnotobiotic mouse model, the addition of narrow host range phages can suppress populations of host bacteria which then indirectly impacts populations of non-host bacteria (Hsu et al., 2018). Bao et al. (2018) found that addition of either lytic or temperate phage monocultures into the gut can change relative abundances of bacterial populations at the phylum and genus level. Some evidence suggests that targeted phage therapy may be used to control specific bacterial pathogens in the gut environment, such as *Enterococcus faecalis* (Bolocan et al., 2019). Direct interactions between fecal VLPs and the host immune system may also shape the gut microbial community (Kernbauer and Cadwell, 2014; Yang et al., 2016; Van Belleghem et al., 2017; Gogokhia et al., 2019).

A HFD, or “Western diet,” is a well-known risk factor for numerous chronic diseases including metabolic dysfunction, cancer, and gastrointestinal disorders (Murphy et al., 2015). Accumulating evidence demonstrates that Western diet-induced dysbiosis is a driving force in the development of these diseases (Cani et al., 2007; Guo et al., 2017). The present study found that diets high in saturated fats can promote SIBO. Findings of SIBO are present in up to 78% of patients with IBS (Pimentel et al., 2000; Ghoshal et al., 2017); the exact contribution of diet to SIBO in these patients is not known. Currently, antibiotics are used to reduce bacterial density of the small intestine in SIBO. This strategy is only effective at eliminating symptoms of SIBO in 48% of patients with IBS and recurrence is common (Pimentel et al., 2000). The present study provides evidence for the intriguing possibility that fecal VLPs may be a potential modifier of the gut microbiome. Although VLPs from HFD donors reduced bacterial density of HFD recipients, this approach has limited clinical relevance since fecal transplants from dysbiotic donors may pose a risk for the recipient (Wong et al., 2017). Autologous FMT treatments have had some success in resolving antibiotic-induced dysbiosis (Suez et al., 2018), therefore autologous fecal VLP transplantation may be a less risky strategy to utilize the locally adapted phage population for treatment of SIBO.

This study demonstrates the biological activity of fecal VLPs during FMT on the recipient gut microbiome. It is clear from our findings that the non-bacterial components transferred during FMT deserve further investigation.

## Data Availability Statement

The datasets generated for this study are available on request to the corresponding author.

## Ethics Statement

The animal study was reviewed and approved by Institutional Animal Care and Use Committee at the New Mexico VA Health Care System following guidelines provided by the Guide for the Care and Use of Animals of the National Research Council of the National Academies.

## Author Contributions

DML, BK, and HL designed research. DML, NR, and AC-P performed research. DML and DL analyzed data. DML, BK, and HL wrote the paper.

### Conflict of Interest

DML, BK, and HL have I. P. rights in related areas. The remaining authors declare that the research was conducted in the absence of any commercial or financial relationships that could be construed as a potential conflict of interest.

## Abbreviations

FMT: Fecal microbiota transplantation
VLP: Virus-like particle
SIBO: Small intestinal bacterial overgrowth
PBS: Phosphate-buffered saline
HFD: High-fat diet
SD: Standard diet.
